# Smoking related attitudes, motives, and behaviors of male secondary school students in an urban setting of China

**DOI:** 10.1186/s40064-016-3694-z

**Published:** 2016-11-28

**Authors:** Xianglong Xu, Cheng Chen, Abu S. Abdullah, Lingli Liu, Manoj Sharma, Yaping Li, Yong Zhao

**Affiliations:** 1 School of Public Health and Management, Chongqing Medical University, Chongqing, 400016 China; 2 Research Center for Medicine and Social Development, Chongqing Medical University, Chongqing, 400016 China; 3 Innovation Center for Social Risk Governance in Health, Chongqing Medical University, Chongqing, 400016 China; 4 Xiangya School of Public Health, Central South University, Changsha, 410001 Hunan Province China; 5 Global Health Program, Duke Kunshan University, Kunshan, 215347 Jiangsu Province China; 6 Duke Global Health Institute, Duke University, Durham, NC 27710 USA; 7 Department of General Internal Medicine, School of Medicine, Boston University Medical Center, Boston, MA 02118 USA; 8 Behavioral and Environmental Health, School of Public Health, Jackson State University, Jackson, MS 39213 USA

**Keywords:** Attitudes, Smoking behaviors, Urban, Male, Secondary school students

## Abstract

**Background:**

Most smokers have their first experience of cigarette use when they are teenagers (i.e. at schools). Aiming tobacco control initiatives at secondary school students offers preventive measures to eliminate or reduce tobacco use initiation by students. This study assessed smoking-related attitudes, motives and behavior as well as other factors associated with smoking among male secondary school students in an urban setting in China.

**Methods:**

A cross-sectional survey was conducted in urban areas of Chongqing using a structured questionnaire administered to 1297 male secondary school students. Logistic regression analysis was employed to investigate the factors that affect smoking.

**Results:**

Of the participants (n = 1297), 27.7% were secondary and 72.3% were middle school students. Overall, 30.5% of the participants were smokers. The majority of the students procured their first cigarette from friends. The motivations of middle school students to smoke their first cigarette were curiosity (70.5%) and imitation (13.1%). The motivations of high school students for smoking their first cigarette were to satisfy their curiosity (51.8%), relief of stress and social pressures (17.1%), and imitation of smoker friends (15.9%). Respondents from poor family financial conditions were more likely to smoke than those from rich family financial conditions [odds ratio (OR) 1.59, 95% confidence intervals (CI) 1.00, 2.52]. Respondents whose fathers were smokers were more likely to smoke than those whose fathers were non-smokers (OR 1.65, 95% CI 1.25, 2.17). Respondents who had one or two smoker friends (OR 2.98, 95% CI 1.93, 4.60) and three or more smoker friends (OR 5.92, 95% CI 4.35, 8.05) were more likely to smoke than those who did not have any. Respondents who were neutral (OR 0.43, 95% CI 0.20, 0.91) and disagreed about the item “friends smoking” (OR 0.18, 95% CI 0.08, 0.39) were less likely to smoke than those who agreed with the item.

**Conclusions:**

This study indicates that male students who have negative attitudes toward smoking, who do not have a father or friends who smoke, and are from affluent backgrounds exhibit low tendency to start smoking at a young age. This study provides some implications for tobacco control policies among male secondary school students in urban settings.

## Background

Tobacco use is becoming one of the major preventable causes of premature death, disease, and disability globally (National Center for Chronic Disease Prevention and Health Promotion and U.S. Office on Smoking and Health 2014). Most of smokers are men, and almost 800 million men smoke, with a third of all smokers living in China (Mackay 2012). Smoking among teenagers is becoming a serious public health problem. Data from the Global Youth Tobacco Survey (GYTS) showed that, 16.2–30.1% teenagers initiated smoking and 8.6–14.6% had smoked (Warren et al. 2000). Approximately 90% of adult smokers began smoking before reaching 18 years old in the US (U.S. Department of Health and Human Services 2012), and approximately 40% of adolescent smokers in China started smoking before reaching 10 years old (Hesketh et al. 2001). In 2012, 52.9% of male were smokers, and 31.0% of male smokers never seriously thought of quitting smoking in China (Xu et al. 2015). Tobacco control among adolescents is necessary and important to prevent many long-term diseases associated with smoking (U.S. Department of Health and Human Services 1994, 2012).

Based on the 2014 China Youth Tobacco Survey Report, overall 6.9% of junior middle and high school students smoked; the prevalence in male students (11.2%) was higher than in female students (2.2%) (China Center for Disease Control and Prevention 2014). A systematic review of smoking initiation among Asian adolescents showed that male youths were more likely to initiate smoking (Talip et al. 2016). Teenagers are in the period of growth and development, when all physiological systems and organs are not yet fully developed; thus, their unique physiological characteristics make them more susceptible to tobacco (Centers for Disease Control and Prevention 1994).

Tobacco exposure in early age indicates that the age of onset of numerous disease processes may be earlier and the incidence may be higher (WHO 2015). In particular, middle school is an important stage of physical and mental development. Cognition and the use of tobacco significantly affect tobacco control and smoking status (Treacy et al. 2007; Guo et al. 2012). The smoke-free campus policy can prevent non-regular smokers in high school from becoming regular smokers in college (Butler et al. 2012). Changing the social environment through policies and decreasing exposure of non-smoking students to tobacco use may decrease smoking initiation (Ridner 2005).

Although numerous studies have documented rates and risk factors of smoking among adults, reliable evidence of those pertaining to youth from China is minimal. Many studies have explored the factors associated with smoking, but smoking initiation and tobacco use among youth in developing countries is rarely addressed. Clarifying the factors that affect smoking among youth is necessary to reduce smoking in China. Considering how smoking cessation poses challenges for many smokers (Lina et al. 2016), encouraging and educating male teenagers to not start smoking tobacco has significant public health benefits. Investigating the association between smoking initiation and relevant characteristics can assist in developing novel tobacco control measures. Smoking prevention among teenagers can also bring lifelong health benefits. This study assessed smoking-related attitudes and behaviors as well as factors associated with smoking initiation among male secondary school students to address the following four research questions:

1. What is the motivation to smoke for the first time?
2. How is the first cigarette procured?
3. What are the related attitudes, motives and behaviors toward smoking?
4. What are the factors that affect smoking?

## Methods

### Participants

The study design and methods, including the inclusion criteria and the exclusion criteria and the population, as well as the survey administration, pilot study and methods of data collection have been reported previously (Xu et al. 2016). In June 2014, a formal investigation was conducted in the main urban areas of Chongqing. Among 1370 respondents, 1297 (94.7%) people answered all the questions, and 73 questionnaires were excluded because of missing data, resulting in a final sample of 1297 for analysis. In the Chinese education system, grades 7–9 comprise junior middle school, and grades 10–12 are high school. Considering the low prevalence of smoking in female students, this study only focused on male students to investigate typical smoking-related characteristics. Research on smoking among youth has traditionally included both middle and high schools students (Holmberg and Hellberg 2007). We obtained written informed consents from all parents or main caregivers of the enrolled students through parent–teacher conferences. The study was conducted in accordance with the Declaration of Helsinki, and the protocol was approved by the Ethics Committee of Chongqing Medical University.

### Questionnaire content

The questionnaire was derived from the 2014 China Youth Tobacco Survey Report (China Center for Disease Control and Prevention 2014). The questionnaire was customized for the target population based on the pilot study, and the final draft of the questionnaire was agreed upon after several discussions with experts and the pilot investigators. The questionnaire was piloted on 50 participants in one of the schools for finalization. We modified the questionnaire based on the results of the pilot study, especially the presentation of questions and the answer options.

### Demographics

Demographic data included age, school status (middle/high school students), smoking status of parents (non-smoker/smoker), family structure (intact family/non-intact family), per capita household income [high (>US$750), average (US$225–$750), and low (<US$225)]. The definition of intact family meant teenagers live with both biological parents, and non-intact family is defined as family with the absence of one or both biological parents.

### Smoking status

Smoking status of students and parents were included. The number of smoking friends and family members were also included. Smoking status was determined by one item which asks respondents whether they were smokers. Current smoker was defined as a person who smoked any form of tobacco at the time of the interview. The method used to assess the smoking status of parents was conducted the same way. Participants were asked the following questions: “Does your father smoke? (yes/no)” and “Does your mother smoke? (yes/no).”

### Smoking-related attitudes

Smoking-related attitudes were determined by their agreement on the following statements: (1) decided not to smoke in the future, (2) proud of being a non-smoker, (3) supported others not to smoke, (4) concerned about my health, (5) had confidence to refuse to smoke, and (6) looked for ways to refuse to smoke or quit smoking.

### Smoking-related behaviors

A few questionnaire items measured their smoking-related behaviors by the responses to “the way to obtain the first cigarette,” “motivation to smoke for the first time,” “supported smoking ban in public places (yes/no),” and “tried to quit smoking (yes/no).”

### The way to obtain the first cigarette

The way to obtain the first cigarette included “friends gave it to me,” “took it from home by myself,” “parents gave it to me,” “bought it by myself,” and “other ways.”

### Smoking motives

The motivation to smoke for the first time included “imitation of smoker friends,” “influenced by film or television,” “rebellious psychology,” “relieve stress and social pressures,” and “other reasons.” Social pressure came from peers, schoolwork, parents, teachers, and ego development. After “other ways” and “other reasons”, blank spaces were provided for students to write related information.

### Data analyses

Participant characteristics were summarized using frequencies, percentages and descriptive analysis (means, standard deviations, and percentages). Chi square tests were used to compare differences in categorical variables, and the *t* tests were used to compare differences in continuous variables. Logistic regression analysis was employed to determine factors that affect smoking among male secondary school students. All statistics were performed using two-sided tests, and statistical significance was considered at p < 0.05. All data were analyzed with Statistical Package for Social Sciences (IBM-SPSS), Version 22.0.

## Results

### Characteristics of participants

The participants included 1297 males, of which 359 (27.7%) were middle school students and 938 (72.3%) were high school students. Among the participants, 395 (30.5%) were smokers. The smoking rate was 35.6% and 17.0% for high and middle schools students, respectively. Compare with middle school students, the smoking rate of high school students was statistically higher than (p < 0.001). The smoking rate of the fathers of smokers was statistically higher than that of fathers of non-smokers (p < 0.001). The differences on maternal smoking status (p = 0.01), socioeconomic status (p < 0.001), and number of smoking friends and family members between smokers and non-smokers (p < 0.05) were statistically significant (see Table 1).

**Table 1:** Characteristics of the sample (n, %)

Variables	Smoker (n = 395)	Non-smoker (n = 902)	p-value
Age (mean ± SD)	16.5 ± 1.4	15.4 ± 1.9	<0.001*
School status			<0.001*
Middle school	61 (15. 4)	298 (33.0)	
High school	334 (84.6)	604 (67.0)	
Family structure			0.336
Intact family	323 (81.8)	760 (84.3)	
Non-intact family	69 (17.5)	139 (15.4)	
Missing	3 (0.8)	3 (0.3)	
Per capita household income			<0.001*
High	57 (14.4)	171 (19.0)	
Average	244 (61.8)	583 (64.6)	
Low	94 (23.8)	148 (16.4)	
Paternal smoking status			<0.001*
Non-smoker	140 (35.4)	449 (49.8)	
Smoker	255 (64.6)	453 (50.2)	
Maternal smoking status			0.007*
Non-smoker	370 (93.7)	874 (96.9)	
Smoker	25 (6.3)	28 (3.1)	
The number of smoking friend			<0.001*
None	80 (20.3)	310 (34.4)	
One or two	257 (65.1)	520 (57.7)	
Three or more	58 (14.7)	72 (8.0)	
The number of smoking family member			<0.001*
None	82 (20.8)	582 (64.5)	
One or two	48 (12.2)	99 (11.0)	
Three or more	265 (67.1)	221 (24.5)	
Attitude towards friends smoking			<0.001*
Agree	47 (11.9)	24 (2.7)	
Neutral	246 (62.3)	323 (35.8)	
Disagree	102 (25.8)	555 (61.5)	
Attitude towards family member smoking			<0.001*
Agree	29 (7.3)	20 (2.2)	
Neutral	168 (42.5)	222 (24.6)	
Disagree	198 (50.1)	660 (73.2)	

* Statistically significant (p < 0.05)

### Smoking-related attitude

A higher proportion of non-smokers (91.1%) than smokers (66.8%) responded that they had “decided not to smoke in the future,” (p < 0.001). Non-smokers had a more negative attitude toward smoking than smokers, and the former was “proud of being a non-smoker” (p < 0.001), “supported others not smoke” (p < 0.001), “concerned about my health” (p < 0.001), “had confidence to refuse smoking” (p < 0.001), and “looked for ways to refuse or quit smoking” (p < 0.001) (see Table 2).

**Table 2:** Smoking-related attitude (n, %)

Variable	Smoker (n = 395)	Non-smoker (n = 902)	p-value
Decided not to smoke in the future			<0.001*
Agree	264 (66.8)	822 (91.1)	
Neutral	94 (23.8)	62 (6.9)	
Disagree	37 (9.4)	18 (2.0)	
Proud of being a non-smoker			<0.001*
Agree	254 (64.3)	783 (86.8)	
Neutral	86 (21.8)	90 (10.0)	
Disagree	55 (13.9)	29 (3.2)	
Supported others not to smoke			<0.001*
Agree	273 (69.1)	777 (86.1)	
Neutral	81 (20.5)	99 (11.0)	
Disagree	41 (10.4)	26 (2.9)	
Concerned about my health			<0.001*
Agree	351 (88.9)	875 (97.0)	
Neutral	28 (7.1)	22 (2.4)	
Disagree	16 (4.1)	5 (0.6)	
Had confidence to refuse to smoke			<0.001*
Agree	294 (74.4)	827 (91.7)	
Neutral	76 (19.2)	61 (6.8)	
Disagree	25 (6.3)	14 (1.6)	
Looked for ways to refuse to smoke or quit smoking			<0.001*
Agree	286 (72.4)	794 (88.0)	
Neutral	75 (19.0)	91 (10.1)	
Disagree	34 (8.6)	17 (1.9)	

* Statistically significant (p < 0.05)

### Smoking-related behaviors

Non-smokers scored a higher percentage of “encouraged others not to smoke (p < 0.001)” and “tried to get parents to quit smoking (p = 0.04)” than smokers. However, no statistically significant difference was observed on “supported smokers to quit smoking” and “Tried to get friends to quit smoking” between non-smokers and smokers (see Table 3).

**Table 3:** Smoking-related practices (n, %)

Variable	Smoker (n = 395)	Non-smoker (n = 902)	p-value
Encouraged others not to smoke			<0.001*
Yes	284 (71.9)	732 (81.2)	
No	111 (28.1)	170 (18.9)	
Supported smokers to quit smoking			0.794
Yes	277 (70.1)	626 (69.4)	
No	118 (29.9)	276 (30.6)	
Tried to get friends to quit smoking			0.293
Yes	276 (69.9)	656 (72.7)	
No	119 (30.1)	246 (27.3)	
Tried to get parents to quit smoking			0.042*
Yes	269 (68.1)	664 (73.6)	
No	126 (31.9)	238 (26.4)	

* Statistically significant (p < 0.05)

### Smoking-related behaviors among smokers

Statistically significant differences were observed on “the way to obtain the first cigarette” (p = 0.001) and “motivation to smoke for the first time” (p = 0.01) between high and middle school students. Middle school students obtained the first cigarette when friends provided it (44.3%), took it from home by themselves (16.5%), and other ways (21.3%). More high school students obtained the first cigarette when friends provided it (62.3%), took it from home by themselves (18.6%), and other ways (8.1%). According to the answers of a few participants, other ways of obtaining the fist cigarette included “stealing it from family members” and “obtaining it from a party with friends”. Among the high school students, 93.4% supported smoking ban in public places, which was statistically higher than 75.2% of middle school students (p = 0.02). A total of 62.3% of middle and 58.6% of high school students tried to quit smoking (p = 0.60) (see Table 4).

**Table 4:** Comparison of motives and behaviors among smokers between high school students and middle school students (n, %)

Variable	Middle school (n = 61)		High school (n = 334)		p-value
	Yes	Rank	Yes	Rank	
The way to obtain the first cigarette					
Friends gave it to me	27 (44.3)	1	208 (62.3)	1	0.004*
Took it from home by myself	10 (16.4)	3	62 (18.6)	2	
Parents gave it to me	6 (9.8)	4	14 (4.2)	5	
Bought it by myself	5 (8.2)	5	23 (6. 9)	4	
Other ways	13 (21.3)	2	27 (8.1)	3	
Supported smoking ban in public places					0.004*
Yes	57 (93.4)	1	251 (75.2)	1	
No	4 (6.6)	2	83 (24.9)	2	
Tried to quit smoking					0.008*
Yes	57 (93.4)	1	196 (58.7)	1	
No	4 (6.6)	2	138 (41.3)	2	
The motivation to smoke for the first time					0.008*
Curious	43 (70.5)	1	173 (51.8)	1	
Imitation of smoker friends	8 (13.1)	2	53 (15.9)	3	
Influenced by film or television	2 (3.3)	4	5 (1.5)	6	
Rebellious psychology	1 (1.6)	5	13 (3.9)	5	
Relieve stress and social pressures	0 (0.0)	6	57 (17.1)	2	
Other reasons	7 (11.5)	3	33 (9.9)	4	

* Statistically significant (p < 0.05)

### Smoking-related motives among smokers

The motivations of middle school students to smoke for the first time were curiosity (70.5%), imitation of smoker friends (13.1%), and other reasons (11.5%). The motivations of high school students to smoke for the first time were to satisfy curiosity (51.8%), relieve stress and social pressures (17.1%), and imitate smoker friends (15.9%). Some students chose other motivations, such as “forced by roommates”, “for recreation and entertainment” (see Table 4).

### Logistic regression model to probe factors that affect smoking

Students who lived within low socioeconomic status were 1.59 times more likely to try cigarettes than students who lived within high or average per capita household income [adjusted odds ratio (OR) were 1.59, 95% confidence intervals (CI) were 1.00, 2.52]. Students whose fathers smoked were 1.65 times more likely to smoke than those whose fathers do not smoke (adjusted OR 1.65, 95% CI 1.25, 2.17). Students who had one to two friends who smoke were 2.98 times more likely to try cigarettes than students who did not have friends who smoke (adjusted OR 2.98, 95% CI 1.93, 4.60), and students who had three or more friends who smoke were 5.92 times more likely to try cigarettes (adjusted OR 5.92, 95% CI 4.35, 8.05). Students who disagreed (adjusted OR 0.18, 95% CI 0.08, 0.39) or those who were neutral (adjusted OR 0.43, 95% CI 0.20, 0.91) with regard to friends smoking were less likely to smoke than those who agree with friends smoking (Table 5).

**Table 5:** Logistic regression model for identifying factors that affect smoking among male secondary students

Variable	OR	95% CI	p-value
Per capita household income			
High	1		
Average	1.25	(0.85, 1.85)	0.255
Low	1.59	(1.00, 2.52)	0.048*
Father smoking status			
Non-smoker	1		0.001*
Smoker	1.65	(1.25, 2.17)	
The number of smoking friend			
None	1		
One or two	2.98	(1.93, 4.60)	<0.001*
Three or more	5.92	(4.35, 8.05)	<0.001*
Attitude toward friends smoking			
Agree	1		
Neutral	0.43	(0.20, 0.91)	0.026*
Disagree	0.18	(0.08, 0.39)	<0.001*
Attitude toward family member smoking			
Agree	1		
Neutral	1.63	(0.67, 3.93)	0.281
Disagree	1.21	(0.50, 2.92)	0.667

*OR* odds ratio, *CI* confidence intervals

* Statistically significant (p < 0.05)

## Discussion

Knowledge, attitudes and practices (KAP) model has been widely used in public health. KAP model is based on the principle that increasing knowledge will result in changed attitudes and practices to minimize the burden of diseases (Mascie-Taylor et al. 2003). For example, a KAP study in Thailand showed the positive influence of increasing community knowledge on controlling and preventing dengue, thereby improving prevention practices (Koenraadt et al. 2006). What’s more, some other surveys adopted KAP model include identifying knowledge gaps, cultural beliefs, and behavioral patterns that may pose barriers to controlling infectious diseases (Krentel et al. 2006; Matibag et al. 2007), design of relevant public health awareness campaigns (Cantey et al. 2010), establishing baseline data for planning and implementation, and evaluating control program.

The rate of tobacco use among high school students is higher than that among middle school students. This survey showed that 30.5% of participants were smokers, in which the smoking rate of high school students was 35.6% whereas the smoking rate of middle school students was 17.0%. A study in the US found that 8.3% of participants were current smokers (Adams et al. 2013), and a recent trial of a smoking prevention intervention study in Germany found less than 8.0% were regular smokers in the intervention group, and 10.1% in the control group (Krist et al. 2016). Compared with other countries, the situation of students smoking in China is heightened. A potential interpretation is that this study focused on male students and the definition that we used to define a smoker (i.e. smoking any cigarette in the past) was broad. A cross-sectional analysis in Canada showed that 28.1% of male students smoke and that one or more family members who smoke were considered as a risk factor (Gregoire et al. 2016).

The prevalence of smoking among male students was lower than that among male adults, with a smoking rate of 52% (Xu et al. 2015). For adults, the time of exposure to tobacco use and smoking was longer, which was considered as a type of social propriety in China. These findings may contribute to the higher smoking rate in male adults than in male students. A previous study also showed that the rate trend of tobacco use significantly increased as the grade level increased in middle school students (China Center for Disease Control and Prevention 2014). The present study shows that non-smokers have a more negative attitude toward smoking than smokers. Another study demonstrated that establishing the right attitude toward tobacco control in middle school students was advantageous to reduce their smoking rate (Guo et al. 2012). Additionally, a previous study showed the combination of family opposed to smoking and peers who discourage smoking were advantageous to quitting smoking (Mak et al. 2012). Cooperation between schools and parents in providing smoking cessations measures is imperative to improve smoking cessation in middle and high school male smokers.

This study investigated the means to procure the first cigarette and motivation to smoke for the first time. Most students obtained their first cigarette from their friends or they took it from their home. This finding is similar to the positive association between smoker friends and smoking tendencies in Iranian high school students (Chaman et al. 2015). Teaching students how to uncompromisingly refuse is necessary when faced with the fist cigarette acquired from their friends. Curiosity also played a key role in the initial motivation. A previous study also found that curiosity significantly improved the identification and classification of which adolescents would experiment with smoking (Nodora et al. 2014). Similarly, the evidence in the present study shows that curiosity was the main reason male adolescents smoked for the first time. The other two important motivations include imitating smoker friends and relief of stress and social pressures.

Factors affecting smoking in secondary schools should be considered in tobacco control efforts. In this study, respondents who were from poor family financial conditions were more likely to smoke than were from rich family financial conditions. This finding is consistent with other findings that showed that tobacco use was strongly associated with adolescents living within a low socioeconomic status (SES) (Bird et al. 2016; Rosen et al. 2014). Adolescents with low SES might have received more negative influence from smoking parents or other household members about smoking and less support for not taking up smoking or in smoking cessation. Tobacco controls on secondary school students should focus on low-income families. This study also found that negative attitudes toward smoking behavior and parents who do not smoke are two factors that prevent adolescent smoking among Chinese secondary school students. Respondents whose fathers are smokers are more likely to smoke than those whose fathers are non-smokers, which further confirm that parents, especially fathers, have a significant influence on the smoking behavior of children (Wilkinson et al. 2008). Parents should set an example in front of their children and quit smoking as soon as possible to persuade children not to become a smoker (Clark et al. 2006).

Respondents who have friends who smoke are more likely to smoke. Therefore, the possibility of smoking increased with the number of friends who smoke, suggesting that the peers easily influence the behavior of students. Many studies have reported that peer pressure and fathers who smoke are important factors which influence the smoking of youth (Passey et al. 2011). Numerous examples of smokers in the environment, especially in the companion of a role model who smoke will increase the odds of the smoking behavior of a teenager (Hawkins et al. 1992; Keefe 1994). Therefore, improving the coping skills of secondary school students against peer pressure is important. This study also found that respondents who were neutral and disagreed with the item “friends smoking” were less likely to smoke than those who agreed. The negative attitudes of students toward smoking make them less likely to smoke because smoking-related attitudes influence smoking behavior. Therefore, strengthening tobacco control publicity is necessary for secondary schools to assist students in establishing the right attitude toward smoking. The future direction of tobacco control among middle school students is focused on smoking-related attitudes and peer education.

The results of this study have a few important implications for school health. First, approximately 30.45% of urban male secondary school students are smokers. Strengthening the work of tobacco control among male high school students is urgent. Comprehensive interventions, including individual, campus, family, community, and government strategies, can be used to reduce vulnerability in Chinese secondary school students. Second, many studies indicated that having friends who smoke had an effect on the smoking status of the student, the registered number of friends who smoke is associated with the smoking status of students, especially in most male students who obtained their first cigarette from their friends. This finding suggests peer pressure and influence from fellow classmates as important factors contributing to the initiation of smoking, and important areas to consider when planning smoking prevention programs. Peer education is necessary to inform male students regarding the method on refusing their first cigarette from friends. Third, a meaningful finding was that a considerable number of male students started to smoke because of curiosity. Therefore, reducing student curiosity on smoking and informing them regarding the harmful effects of smoking can play a pivotal role in future research. Fourth, students within low socioeconomic status are more likely to smoke, which suggests the need to adopt a protocol for screening and education by school nurses and teachers with regards to this student population. Moreover, negative attitudes toward smoking behavior were associated with the smoking behavior among male students. The current findings suggest that a comprehensive school-based tobacco control campaign is urgently required.

This study also has some limitations. First, 72.3% of the subjects are high school students, and only 27.7% of the subjects are middle school students, thereby affecting the representativeness of secondary school students. Therefore, this study requires cautious interpretation of the results, especially among middle school students. Second, only urban secondary students were investigated and rural secondary students were not investigated. In China, the smoking rate of rural secondary school students was higher than that of urban secondary school students (Butler et al. 2012). Replicating this study in rural secondary school students would be useful to assess the difference between the urban and rural students in China. Cautions will also be necessary to extrapolate the findings to students in other countries, given the socio-cultural differences between China and elsewhere. Finally, this study was cross-sectional and causal association cannot be confirmed. Moreover, the current study did not assess the tobacco use among female students, therefore, cautions will be necessary to extrapolate the findings to female students.

## Conclusions

Overall, approximately 30.5% of urban male secondary school students were smokers. Students procured their first cigarette primarily from a friend, and the motive to smoke the first cigarette was curiosity. Students who started smoking at a young age were those who had positive attitudes towards smoking, had fathers who were smokers, had a smoker friend, and belonged to poor economic background. Comprehensive interventions, including individual, campus, family, community, and government strategies, can target risky behaviors in an effort to reduce smoking among Chinese secondary school students. These findings enhance the understanding of the epidemic of smoking among male secondary school students in an urban setting of China and should be incorporated into future youth tobacco control strategies.
